# Proteomic Analyses of the Unexplored Sea Anemone *Bunodactis verrucosa*

**DOI:** 10.3390/md16020042

**Published:** 2018-01-24

**Authors:** Dany Domínguez-Pérez, Alexandre Campos, Armando Alexei Rodríguez, Maria V. Turkina, Tiago Ribeiro, Hugo Osorio, Vítor Vasconcelos, Agostinho Antunes

**Affiliations:** 1 CIIMAR/CIMAR, Interdisciplinary Centre of Marine and Environmental Research, University of Porto, Terminal de Cruzeiros do Porto de Leixões, Av. General Norton de Matos, s/n, 4450-208 Porto, Portugal; danydguezperez@gmail.com (D.D.-P.); amoclclix@gmail.com (A.C.); tiago.amribeiro8@gmail.com (T.R.); vmvascon@fc.up.pt (V.V.); 2 Biology Department, Faculty of Sciences, University of Porto, Rua do Campo Alegre, s/n, 4169-007 Porto, Portugal; 3 Department of Experimental and Clinical Peptide Chemistry, Hanover Medical School (MHH), Feodor-Lynen-Straße 31, D-30625 Hannover, Germany; aara259@gmail.com; 4 Division of Cell Biology, Department of Clinical and Experimental Medicine, Linköping University, SE-581 85 Linköping, Sweden; maria.turkina@liu.se; 5 Instituto de Investigação e Inovação em Saúde- i3S, Universidade do Porto, Rua Alfredo Allen, 208, 4200-135 Porto, Portugal; hosorio@ipatimup.pt; 6 Ipatimup, Institute of Molecular Pathology and Immunology of the University of Porto, Rua Júlio Amaral de Carvalho, 45, 4200-135 Porto, Portugal; 7 Department of Pathology and Oncology, Faculty of Medicine, University of Porto, Al. Prof. Hernâni Monteiro, 4200-319 Porto, Portugal

**Keywords:** cnidarian, sea anemone, proteins, toxins, two-dimensional gel electrophoresis, MALDI-TOF/TOF, shotgun proteomic

## Abstract

Cnidarian toxic products, particularly peptide toxins, constitute a promising target for biomedicine research. Indeed, cnidarians are considered as the largest phylum of generally toxic animals. However, research on peptides and toxins of sea anemones is still limited. Moreover, most of the toxins from sea anemones have been discovered by classical purification approaches. Recently, high-throughput methodologies have been used for this purpose but in other Phyla. Hence, the present work was focused on the proteomic analyses of whole-body extract from the unexplored sea anemone *Bunodactis verrucosa*. The proteomic analyses applied were based on two methods: two-dimensional gel electrophoresis combined with MALDI-TOF/TOF and shotgun proteomic approach. In total, 413 proteins were identified, but only eight proteins were identified from gel-based analyses. Such proteins are mainly involved in basal metabolism and biosynthesis of antibiotics as the most relevant pathways. In addition, some putative toxins including metalloproteinases and neurotoxins were also identified. These findings reinforce the significance of the production of antimicrobial compounds and toxins by sea anemones, which play a significant role in defense and feeding. In general, the present study provides the first proteome map of the sea anemone *B. verrucosa* stablishing a reference for future studies in the discovery of new compounds.

## 1. Introduction

Cnidarians represent the largest source of bioactive compounds, as candidates for pharmacological tools [1] and even new drugs for therapeutic treatments [2,3,4]. Unlike toxin from terrestrial animals, cnidarian venoms have not received as much scientific attention [5]. Each one of the around 11,000 living species [6] possess nematocysts [7], which is the organ specialized in the production, discharge and inoculation of toxins [8]. Hence, the toxic feature can be theoretically ascribed to all the members of this Phylum, since nematocysts are the only ones of the three categories of cnidae found in all cnidarians [8]. However, without including components of the venom described at the transcriptomic level, only about 250 compounds have been reported until 2012 [9], although this figure has not increased significantly at the proteomic level in the last five years. The venom of cnidarians is composed mainly by peptides, proteins, enzymes, protease inhibitors and non-proteinaceous substances [9].

Most of the known toxins from cnidarians belong to the Order Actiniaria, Class Anthozoa (sea anemones) [10,11,12,13,14,15,16,17,18,19,20,21,22,23,24,25,26,27,28,29,30,31,32,33,34,35,36]. Among sea anemones, around 200 non-redundant proteinaceus toxins have been recognized to date, including proteins and peptides [32,37]. In addition, another 69 new toxins were revealed by transcriptomic-based analyses, although an additional set of 627 candidates has been proposed comprising 15 putative neurotoxins [38] and 612 candidate toxin-like transcripts from other venomous taxa [39]. In general, peptide toxins from sea anemones can be classified as cytolysins, protease inhibitors or ion channel toxins (neurotoxins), mainly voltage-gated sodium (Na_v_) channel toxins and voltage-gated (K_v_) potassium channel toxins [9,35,40,41,42]. Sea anemones are good candidates as a source of peptide/protein toxins, partly because their toxins are considerably stable compared to other cnidarian toxins (e.g., jellyfish). Only a limited number of sea anemones, however, have been examined for peptide/protein toxins [35], although more than 1000 species have been recorded [43]. Thus, sea anemones represent a relatively unexplored potential source of bioactive/therapeutic compounds.

**Figure 1:**
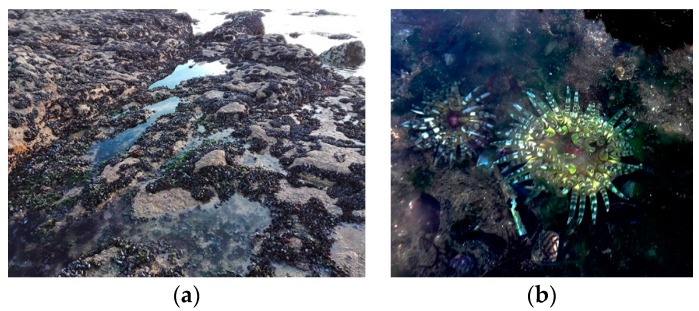
Sampling site at Praia da Memória, Porto, Portugal: (**a**) Picture of tide pools in rocks where inhabits the species of interest *Bunodactis verrucosa*. Note the remained pools at low tide and the relative abundance of mussels in the intertidal community; (**b**) Picture of two individuals of *B. verrucosa* from the sampling site.

The *B. verrucosa* is one of the most common species of sea anemones in the intertidal zone (Figure 1) of Portugal coast [44], yet its proteome, including peptide toxins, remains unexplored. The main goal of the present study was to establish a general proteomic analysis of whole-body extracts from the sea anemone *B. verrucose*; a species known to occur in the northeastern Atlantic Ocean, the North Sea and the Mediterranean Sea [45]. The specimens used in this study came from Portugal coast. The combination of shotgun analyses and two-dimensional gel electrophoresis yielded several proteins, including potential toxins. Until now, just a few chemical studies have been reported from this organism. In fact, to the best of our knowledge, this study provides the first proteomic profile of this species. Most of the proteins identified constitute first report for this species.

## 2. Results and Discussion

### 2.1. Two-Dimensional Gel Electrophoresis and MALDI-TOF/TOF Analyses

**Figure 2:**
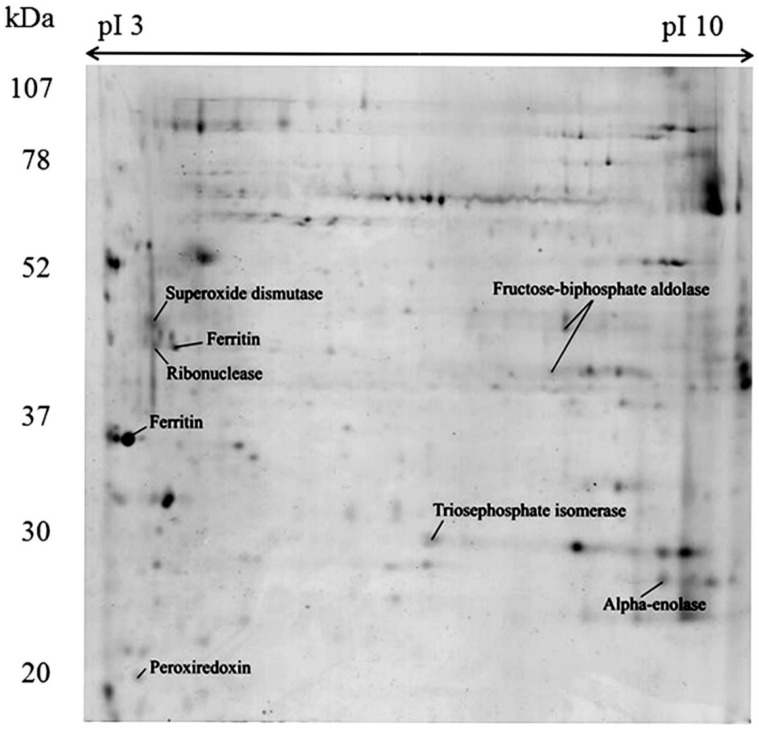
Two-dimensional gel electrophoresis and identification of soluble proteins from the whole-body aqueous extract of *Bunodactis verrucosa*. The first-dimension separation was carried out on 17 cm, pH 3–10 IEF gel strips and the second dimension on 12% sodium dodecyl sulfate-polyacrylamide gel electrophoresis (SDS-PAGE) gels. Gels were stained with colloidal Coomassie blue G-250. Identified proteins are indicated with their most commonly used name.

The gel-based proteome analysis revealed 61 and 36 spots from the soluble fraction (SF) and insoluble fraction (IF), respectively. From the spots analyzed by Matrix-assisted laser desorption/ionization time-of-flight (MALDI-TOF/TOF), 23 peptide sequences belonging to eight proteins were identified in the SF, approximately 38% of the total analyzed (Figure 2, Table 1). Proteins identified in the SF comprised five different enzymes: Superoxide dismutase, Triosephosphate isomerase, Ribonuclease, two Fructose-bisphosphate aldolases and Alpha-enolase. In addition, Peroxiredoxin and two Ferritins were identified. However, three of these proteins matched to “predicted protein” as best hit, but were then further annotated using blastp algorithm in the NCBI with the accession number retrieved from the custom sea anemones databases. Unlike shotgun proteomics, for gel-based analysis were used only two sea anemones databases, since additional search was carried against UniProtKB/Swiss-Prot in the Metazoa section. However, best results corresponded to local analysis. On the other hand, no proteins were identified with statistic confidence from the IF (Figure S1) and in both cases SF and IF, the use of different database like UniProtKB/Swiss-Prot did not improved the identification. The details of blast search and protein identification by MALDI-TOF/TOF mass spectrometry of the protein identified from the 2DE is shown in Table 1. It is noteworthy, that some of the proteins identified have been previously reported in other cnidarians [46,47,48], but constitute the first report in *B. verrucosa*.

The identification rates obtained for SF are similar to those reported in previous studies of other marine species, when comparable proteomics protocols were used [49,50,51]. On the other hand, the absence of identifications in IF is an evidence that our proteomics protocol is likely not optimized for the analysis of the type of proteins present in this fraction. Since IF may be enriched with hydrophobic membrane proteins, the lack of identifications may be related, among other possible causes, to incomplete separation of proteins and to the inefficient digestion of these proteins with trypsin; thus, hindering the generation of a sufficient number of proteolytic peptide fragments for Mass Spectrometry/Mass Spectrometry (MS/MS) sequencing analysis. This limitation of trypsin when cleaving such proteins particularly in the hydrophobic and transmembrane domains can be overcome by combining the activities of other proteases [52,53].

The identified proteins seem to play important roles related with RNA degradation, glycolysis and antioxidant pathways. Moreover, some proteins like alpha aldolase seem to play diverse molecular and physiological roles. In fact, several antibacterial, antiparasitic, antifungical and autoantigen activities have been proposed [54]. Alpha aldolase expression and activity have been associated with the occurrence and metastasis of cancer, as well as with growth, development and reproduction of organisms [54]. Its expression seems to be related to heat shock [55], but it is also probably active under anaerobic condition [54]. In general, some of these proteins act as stress protein against environmental changes by exerting a protective effect on cells.

Ribonucleases, also known as RNases, are common and widely distributed catalytic proteins among animals, involved in the RNA degradation [56]. Three different RNases were detected: triosephosphate isomerase, Fructose-bisphosphate aldolase and Alpha-enolase, which are involved in the glycolytic pathway. Triosephosphate isomerase is a glycolytic enzyme that catalyzes the interconversion of the three-carbon sugars such as dihydroxyacetone phosphate and d-glyceraldehyde 3-phosphate [57]. Aldolases are stereochemistry-specific enzymes acting in a diverse variety of condensation and cleavage reactions [54]. Specifically, fructose-1,6-bisphosphate aldolase is involved in gluconeogenesis and glycolysis, controlling the production of fructose-1,6-bisphosphate from the condensation of dihydroxyacetone phosphate with glyceraldehyde-3-phosphate [58,59]; while Alpha-enolase is a versatile metalloenzyme, that catalyzes the conversion of 2-phosphoglyceric acid to phosphoenolpyruvic acid [54].

On the other hand, Ferritin is one of the most important proteins in iron metabolism, acting as primary iron storage protein or iron transporter, solubilizing iron and thus regulating its homeostasis [60,61]. Peroxiredoxin, also called thioredoxin peroxidase or alkyl hydroperoxide reductase, has been proposed as antioxidant protein [62,63,64]. Both proteins, seem to play an important role by protecting the cells against reactive oxygen species [65], so they are likely to be natural anti-Ultraviolet (UV) radiation agents [66]. Similarly, superoxide dismutase is another relevant antioxidant protein [65,67]. The high expression of this protein as part of the antioxidant defense system makes sense, since aerobic organisms need to deal with oxygen species produced as a consequence of aerobic respiration and substrate oxidation [67].

### 2.2. Protein Identification from Shotgun Proteomics Analysis

A methodology based on shotgun analysis was employed to investigate the whole-body proteome of *B. verrucosa*. This methodology has been previously reported as suitable for diverse purposes related to protein identification such as characterization of complex sample, inference of the main enzymatic pathway involved in a tissue, even to reveal venom composition [68,69,70,71]. Altogether, 688 peptide sequences were identified among the two replicates of the fractions analyzed (SF and IF), which accounted for 412 groups of non-redundant proteins (), retrieved from custom cnidarians databases. Of all protein detected, 97 were identified from two or more peptides. Only four proteins were detected as potential contaminants in the first search against custom database, while 69 sequences accounted for 35 putative proteins as contaminants against UniProtKB/Swiss-Prot database (Table S2). Of such contaminants, 10 proteins were identified from two or more peptides and were related mostly to human keratin and trypsin. In the case of contaminants, proteolytic fragments from trypsin and keratin were the most commonly found, which are difficult to avoid and thus are ubiquitous in proteomic analysis [72]. The functional annotation of all proteins (except for contaminants) was further addressed.

The fact that several IF proteins were identified by this shotgun method shows the increased potential of this method over 2DE/MALDI-TOF/TOF for the analysis of membrane proteins, even when carried out based on the activity of a single protease (trypsin).

All proteins identified from the gel-based analysis were also found among those identified by the shotgun proteomic analysis. As an example, the shotgun analysis allowed the identification of Peroxiredoxin (XP_001640260.1, see Table 1) from two peptides sequences belonging to different organisms (Table S1): one peptide matched Peroxiredoxin-4 (KXJ19217.1) from *E. pallida*, and the second one Peroxiredoxin-4 (KXJ22794.1) from *E. pallida* and peroxiredoxin-like isoform X2 (XP_015769163.1) from *A. digitifera*. In the case of Peroxiredoxin-4 (KXJ22794.1), four peptides were identified for the protein and four for the protein groups (see razor + unique, terms_description in Table S1). However, only nine peptides generated by MALDI-TOF/TOF fragmentation from gel spots, were also detected within peptides resulting from the Orbitrap’s approach. Despite the smaller number of protein identified from 2DE gel, this methodology represented a complement for shotgun proteomics analysis, increasing the number of peptides for the reconstruction of each protein. In fact, in 2D-MALDI fingerprint approach the number of peptides matching some proteins such as superoxide dismutase (KXJ18609.1), alpha-enolase (XP_001632906.1), triosephosphate isomerase (XP_001633516.1) and both fructose-bisphosphate aldolase (XP_001629735.1; XP_001629735.1), were identified with higher confidence in gel-based analyses than in the shotgun methodology.

### 2.3. Protein Gene Ontology Annotation

**Figure 3:**
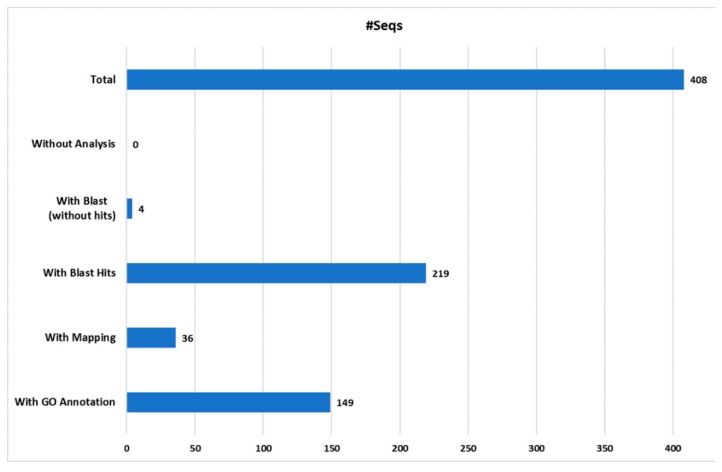
Blast2Go data distribution chart. The number of sequences (**#**Seqs) analyzed and annotated with Blast2Go software from the four custom cnidarian databases used.

The proteomics identification pipeline using the Maquant software and 4 sequence databases, retrieved mostly “predicted” protein products. Therefore, these sequences were further blasted and mapped using the Blast2Go software (version 2.4.4) [73], (Figure 3). From a total of the 412 proteins identified with Maxquant software, 408 were successfully mapped using the Blast2Go software (Figure 3). The remaining four proteins, which were not submitted to further analysis, corresponded to potential contaminants. Out of the total number of proteins analyzed (408), 149 proteins were successfully annotated, representing the 36.5%. Thus, 259 proteins remained without Gene Ontology (GO) annotation, of which only four proteins were blasted without hits, 36 were mapped and 219 yielded positive hits. In total, 223 proteins were not included into the GO annotation considering the level 2 of protein classification, likely due to the absent of similar protein sequences in the protein databases. Moreover, most of these proteins retrieved as hits from cnidarian databases were “predicted”. This result confirms the limited information known about sea anemones and cnidarians products.

**Figure 4:**
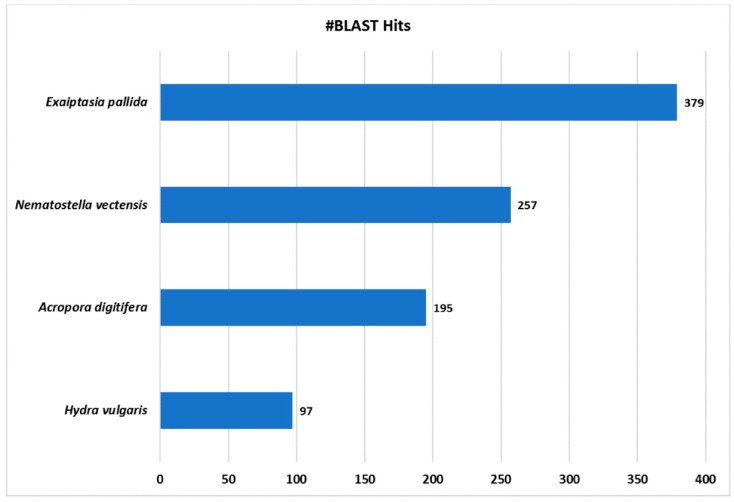
Blast2Go Species distribution chart. Number of blast hits (#BLAST Hits) retrieved are shown from the four cnidarian databases analyzed.

Among the four databases analyzed, most hits corresponded to the species *E. pallida*, followed by *N. vectensis* (Figure 4), as expected according to its relative phylogenetic position [74], although *E. pallida* has the largest number of proteins among the databases used. Afterwards, the proteins identified as positive hits were functionally annotated per the GO nomenclature. Then, GO terms were assigned to each contig and annotated per GO Distribution by Level (2), regarding the three major GO categories: Biological Process (BP), Molecular Function (MF) and Cellular Components (CC).

**Figure 5:**
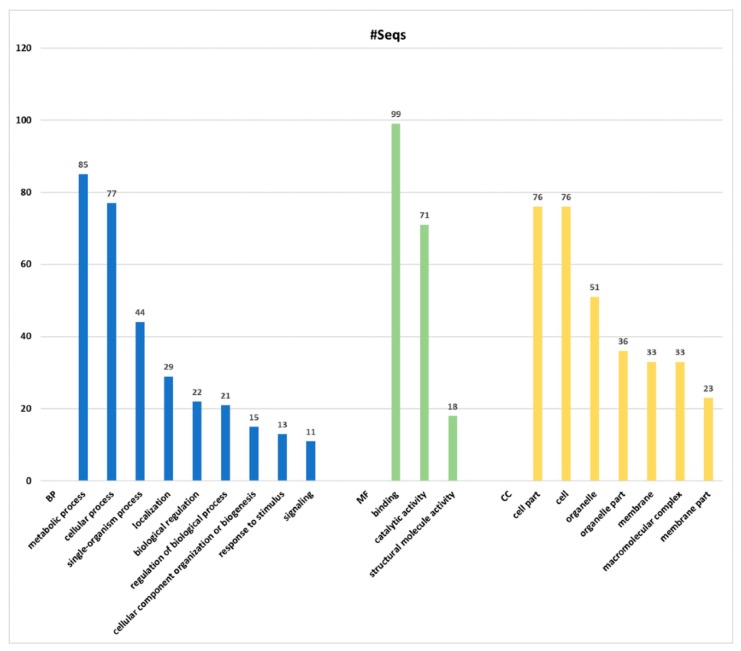
Blast2Go hits Gene Ontology (GO) annotation. Number of sequences (**#**Seqs) corresponding to blast hits annotation are based on the three major GO Categories of GO Distribution by Level (2): Biological Process (PB) in blue, Molecular Function (MF) in green and Cellular Components (CC) in yellow.

The groups of proteins obtained from high-throughput analyses were classified per Blast2Go software, considering the GO Distribution by Level (2) (Figure 5). The most represented GO terms in the category of BP were metabolic process (GO:0008152), followed by cellular process (GO:0009987) and single-organism process (GO:0044699). In the case of MF, the most matched GO terms were binding (GO:0005488), catalytic activity (GO:0003824) and structural molecule activity (GO:0005198), in this order; whereas in the category of CC the most significant were cell part (GO:0044464), cell (GO:0005623) and organelle (GO:0043226). It is noteworthy that some proteins can be included in more than one GO term, since each protein could play diverse roles. Thus, some ambiguities can be found in the proteins reported for each category; and also, the total number of protein may apparently be overestimated. Details of GO annotation and protein accession number can be found on Figure S2 and Table S3.

Among the 111 proteins matching to the GO term BP, 85 proteins (76.56%) classified as metabolic process, 77 (69.37%) for cellular process and 44 (39.64%) as single-organism process. In this group, in the GO level 3, 64 proteins were related with the GO name of “primary metabolic process” and “organic substance metabolic process”, both belonging to “metabolic process” as parent. Besides, 52 proteins were associated with “cellular metabolic process”, which were involved in both metabolic process and cellular process as parents (for details of GO annotation see Figure S2, Table S3).

In total, 86 proteins were included in the category of the CC. Among them, 76 proteins (88.37%) matched for “cell part” and “cell”. However, this is an ambiguity, since all sequences detected as “cell part” are part of the “cell” category (Figure S2). Although, other proteins represented by the sublevels, related to cytoplasmic elements as part of intracellular components, were also subcategories of the “cell”. The GO “intracellular” was more represented with 73 proteins (84.88%) in level 3 than those “organelle” and “membrane” in the superior level 2, with 51 proteins (59.3%) and 33 proteins (38.37%), respectively.

In addition, 135 proteins were grouped into the MF category. Among them, “binding” with 99 proteins (73.3%) was the most significant one. In this group, a total of 66 (48.89%) proteins were involved in “ion binding”, whereas both “heterocyclic compound binding” and “organic cyclic compound binding” hit 62 proteins (45.93%). The second most significant GO term “catalytic activity” comprised 71 proteins (52.59%), of which the most remarkable function was “hydrolase activity”, accounting for 37 proteins (27,41%) acting mainly on acid anhydrides, in phosphorus-containing anhydrides. Moreover, 18 of these enzymes were involved in pyrophosphatase activity, of which 17 are associated with nucleoside-triphosphatase activity (Figure S2).

### 2.4. Top KEGG Pathways

On the other hand, the Kyoto Encyclopedia of Genes and Genomes (KEGG) analyses revealed 28 enzymes involved in 41 different pathways. The accession number of the protein involved in each pathway and other details can be found in Table S4. Considering the number of proteins matched, the most relevant pathways were Purine and Thiamine metabolism, with 18 and 17 proteins matched, respectively (Table 2). In addition, three enzymes: adenylpyrophosphatase, phosphatase and RNA polymerase were found to be involved in the Purine metabolism pathway, whereas only a phosphatase resulted in the Thiamine metabolism. The Purine metabolism pathway is close related to the metabolism of nucleotide [75], since purine constitutes subunits of nucleic acids and precursors for the synthesis of nucleotide cofactors, whereas Thiamine metabolism pathway is fundamental in the metabolism of carbohydrates [76].

Interestingly, one of the most significant among the top twenty pathways was the biosynthesis of antibiotics. In that pathway, a total of 14 proteins, accounted for 13 enzymes grouped into five major families: dehydrogenase, transaminase, carboxykinase (GTP), hydratase, isomerase and aldolase. Most proteins matched in this pathway belong to the larval stage of *N. vectensis*. This result is particularly interesting, because of the abundance of proteins involved in defenses against pathogens, during the most vulnerable stage in the animal life cycle. Thus, this finding supports that sea anemones may be considered as a promising source of antibiotic compounds [77,78,79]. Other relevant pathways were glycolysis/gluconeogenesis and carbon fixation in photosynthetic organisms, both involved in the production of energy. The presence of proteins associated with carbon fixation in photosynthetic organisms is likely due to symbionts such as zooxanthellae, considering to be present in sea anemones [80,81].

The isomerase detected in the biosynthesis of antibiotics pathway, was the same to that identified in the gel-based analyses as triosephosphate isomerase from *N. vectensis* (XP_001633516.1). This one is also involved in other pathways such as glycolysis/gluconeogenesis, carbon fixation in photosynthetic organisms, fructose and mannose metabolism and inositol phosphate metabolism. The predicted protein (XP_001632906.1), homologue to alpha-enolase, and the fructose-bisphosphate aldolase (XP_001629735.1) from *N. vectensis*, were both involved in the pathways of biosynthesis of antibiotics and glycolysis/gluconeogenesis. In addition, the mentioned predicted protein was also found in the methane metabolism pathway, while the fructose-bisphosphate aldolase also occurred in some pathways such as carbon fixation in photosynthetic organisms, methane metabolism, pentose phosphate pathway and fructose and mannose metabolism. In general, these analyses support the diverse roles of some of the proteins identified, given additional information related to its biological function.

### 2.5. Detection of Potential Toxins

Among all peptides detected, 63 sequences matched for 58 potential toxins (Table S2), but only five toxins with more than one peptide (Table 3). Specifically, the five proteins matched as potential toxins were retrieved from different species other than cnidarians and each was reconstructed from two peptide sequences. Besides, these peptides were not redundant to those proteins reconstructed from the previous analyses with the four cnidarians database. In fact, the origin of such peptides by fragmentation of the protein matched as potential toxin (Table 3), which represents a better explanation for our results. Therefore, it is unlikely a false-positive assumption that the peptides were generated from proteins related to potential toxins.

The proteins identified as potential toxins comprise several previously reported toxins and other non-reported in cnidarians. Herein, we found two proteins related to metalloproteinases, one zinc metalloproteinase/disintegrin (VM2M2_DEIAC) of the snake *Deinagkistrodon acutus* [82] and another one called neprilysin-1 (NEP_TRILK) from brush-footed trapdoor spider *Trittame loki* [83]. Both proteins represent two of the three classes of metalloproteinases found in the hydra genome: astacin class, matrix metalloproteinase class, and neprilysin [84]. Metalloproteinases have been subsequently reported in hydra [85,86], jellyfish [9,87,88], but less in sea anemones [89]. Their structure and function seem relatively conserved among metazoans [87], since they can play a broad range of roles in biological process related to hydrolytic functions and development [84]. However, the peptides obtained matched specifically to proteins, which have been proposed as venom components [82,83]. In general, the most significant role of these protein (zinc metalloproteinase/disintegrin, neprilysin-1), must be related to its capacity of breakdown the extracellular matrix [84]. Moreover, this protein displays gelatinolytic and fibrinolytic activities, as previously reported from the venoms of four Scyphozoan jellyfishes [88].

Another protein detected matched to a phospholipase A2 (PLA_2_) called vurtoxin (PA2B_VIPRE) from the steppe viper *Vipera renardi* [90]. Phospholipases A2 are commonly found in the venom of the most toxic animals like cnidarians, cephalopods, insects, arachnids, and reptiles [91]. Specifically, vurtoxin showed homology with the neurotoxic PLA_2_ ammodytoxins [90]. However, it is not clear if this toxin can act as neurotoxin in this species, since vurtoxin occurred as a minor component in the venom of *V. renardi* [90]. In general, the biological role of PLA2_s_ could be diverse. PLA_2_s can act in the arachidonic pathway or in the calcium-dependent hydrolysis of the 2-acyl groups in 3-sn-phosphoglycerides, showing a preference for phosphatidylglycerol over phosphatidylcholine [92,93]. This biological role prevails in cnidarians, showing a significant phylogenetic distance to higher metazoans PLA_2_s, been proposed as the ancestors [94]. On the other hand, the biological activity of PLA_2_s in reptiles has been revealed most as antiplatelet, myotoxic, and neurotoxic [93,95].

In addition, two putative neurotoxins named as alpha-latroinsectotoxin-Lt1a (LITA_LATTR) and SE-cephalotoxin (CTX_SEPES) were identified. The first one, also known as alpha-LIT, was purified from venom glands of the Mediterranean black widow spider *Latrodectus mactans tredecimguttatus* [87]. The proposed mechanism of toxicity involved presynaptic effects, acting selectively only for insects [87]. On the other hand, SE-cephalotoxin has been characterized from the salivary gland of cuttlefish *Sepia esculenta* [88]. The lethality of this toxin was very high to crab, seemingly by neurotoxic mechanism, since the symptoms caused loss of movement, flaccid paralysis and even death [88]. However, SE-cephalotoxin has been considered as a new class of proteinaceous toxin, due to the lack of homology with any other toxins, even those cephalotoxins from octuposes [88]. Therefore, the evidences of a potential SE-cephalotoxin from *B. verrucosa*, constitutes a highlighted finding as the first report of this toxin in sea anemones.

Furthermore, others 53 non-redundant peptide sequences matched to 53 potential toxins, but with only one peptide identified for each protein (Table S2). Of all, 21 peptides sequences matched to 21 potential neurotoxins comprising presynaptic and postsynaptic toxins like ion channel blockers, mostly voltage-dependent potassium and calcium channels. Among them was found a Kunitz-type serine protease inhibitor, which can act as inhibitor of both serine proteases and voltage-gated potassium channels (Kv) [89]. Besides, three metalloproteases, two hyalunoridases, and a Beta-fibrinogenase were detected. On the other hand, seven PLA_2_s and three PLA_D_ occurred within potential toxins. Another potential toxin identified with PLA_2_s activity, was the Helofensin-1 characterized from the genus *Heloderma* [96,97]. This toxin has no hemorrhagic nor hemolytic activities, instead directly inhibited the electrical stimulation of the isolated hemi-diaphragm of mice [96]. Finally, four hemolitic/cytolytic proteins and five additional proteins involved in the coagulation pathway (including two “snaclec”) were found.

### 2.6. Putative Use of Toxins by B. verrucosa in Prey Catching and Feeding

**Figure 6:**
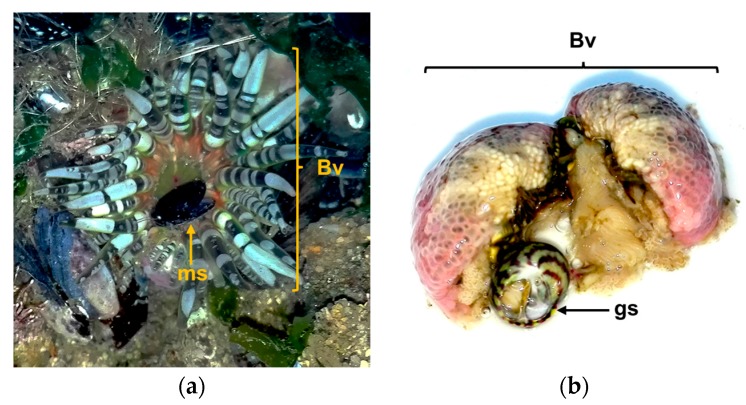
Evidence found relating the *Bunodactis verrucosa* (Bv) feeding on mollusks: (**a**) Specimen of *B. verrucosa* regurgitating an empty mussel’s shell (ms) after body squeezing; (**b**) Gastropod (gs) found into the gastrovascular cavity of *B. verrucosa* after its body dissection.

Sea anemones are ancient active predators, belonging to what is considered “the oldest extant lineage of venomous animals” [98]. The *B. verrucosa* inhabits tidepools in rocks, crevices in shallow water [99], where occurs mussels, gastropods, small crabs, and goby fishes as potential preys. This sea anemone feeds on mussels and small gastropods at least, since we found specimens regurgitating one or more empty mussel shells, after removal from the substrate during sampling (Figure 6). Moreover, we found some specimens containing mussels’ shells and gastropods into the gastrovascular cavity. It is noteworthy that in the sampling area mussels were abundant covering rocks, even in the pools where sea anemones grow (Figure 1). Therefore, these bivalves may constitute the main food source for *B. verrucosa*. This is not an isolated fact, since mussels seem to be the main food source of other intertidal sea anemones like *Anthopleura elegantissima* and *Anthopleura xanthogrammica* [100,101]. Moreover, mussels are suitable to be fed by sea anemones in home aquariums [102]. However, bivalves can close their valves for prolonged periods of time under adverse environmental condition [103,104]. In other words, how can sea anemones obtain nourishments from mussels, if these bivalves tightly close the valves when feel the predator attack?

Mussels are abundant in the intertidal community (Figure 1) and their movements are limited. In this scenario, sea anemones can capture a close mussel with its tentacles and introduce it into the gastrovascular cavity. Once the mussel is captured, it immediately closes its valves and stops filtering. Nonetheless, the sea anemones have cnidocytes in the gastrovascular cavity [8] capable of breaking mussels’ protection. First, hydrolytic enzymes like zinc metalloproteinase/disintegrin, hyaluronidases and proteases found in *B. verrucosa* may be poured into the gastrovascular cavity. The combination of such enzymes could degrade the tissues that seals the shell, probably a dorsal elastic proteinaceous-ligament extending for the length of the hinge [105]; or through the ventral margin of the mussel. The tissues degradation by metalloproteinases can facilitate the diffusion of neurotoxins inside the prey. Then, neurotoxins could act on the adductor muscle, whose loss of function will lead to valve opening.

Specifically, SE-cephalotoxin can diffuse inside the valves, inhibiting the adductor muscles, thus producing flaccid paralysis increasing the valves gape aperture. The high solubility previous reported for SE-cephalotoxin seems to play an important role in the diffusion of this toxin in sea water. This property should be useful whether preys are nearby the sea anemone, because SE-cephalotoxin could disperse around or in the sea water remnant inside the shell after enclosed its valves. Besides, this feature can be used as an advantage to subdue prey prior to eating. Other neurotoxins detected, and the PLA_2_ vurtoxin, are also able to block the adductor muscles. However, the diversity of toxins found is likely related to others potential preys as crabs and goby fishes (Gobiidae, Perciformes), polychaetes worms and starfish. Interestingly, other cephalotoxins have been previously purified from species of octopodiform cephalopods [106,107,108,109], which are likely used to neutralize crabs and bivalves. Altogether, toxins found seemingly act synergistically to subdue mussels. Indeed, a similar mechanism in which hydrolytic enzymes like metalloproteinase facilitate the access of neurotoxic peptides to synaptic targets was previously proposed for the spider *T. loki* [83].

## 3. Materials and Methods

### 3.1. Protein Extraction

Specimens of *B. verrucosa* were sampled at Praia da Memória, Porto, Portugal (Lat/Long WGS84; 41°14′00.0′′ N 8°43′27.0′′ W). Then whole animal bodies (four specimens) were kept at −80 °C, freeze dried and subsequently homogenized in a blender until obtaining a dry powder. Lyophilized material of *B. verrucosa* (0.1 g) was mixed with 500 µL Tris-HCl (40 mM), MgCl_2_ (5 mM), Dithiothreitol (DTT) (1 mM), protease inhibitors (87,785, Thermo Scientific, Waltham, MA, USA), at pH 8.0, (buffer 1) in vortex (2 × 30 s). The mixture was centrifuged at 16,000× *g*, during 20 min at 4 °C. The supernatant (soluble protein fraction, SF) was stored at −20 °C and the pellet was homogenized with 500 µL urea (7 M), thiourea (2 M), CHAPS (4%, *w*/*v*), dithiothreitol (65 mM) and ampholytes (0.8%, *v*/*v*), at pH 4–7 in vortex (2 × 30 s) and incubated overnight, at 4 °C. The homogenate was centrifuged at 16,000× *g*, during 20 min at 4 °C, and the supernatant (insoluble protein fraction, IF) collected and stored at −20 °C. Total protein concentration was estimated according to the Bradford method [110].

### 3.2. Two-Dimensional Gel Electrophoresis

Two-dimensional gel electrophoresis (2DE) was performed as described previously [49]. Duplicate IF and SF (~400 µg of protein) were diluted to 300 µL urea (7 M), thiourea (2 M), CHAPS (4%, *w*/*v*), dithiothreitol (65 mM) and ampholytes (0.8%, *v*/*v*), at pH 4–7 and loaded onto 17 cm, pH 4–7 immobiline dry strips (Bio-Rad, Hercules, CA, USA) with active hydration (50 Volt) for 12 h. Proteins were separated by isoelectric focusing (IEF) in a Protean IEF cell (Bio-Rad) with the following program: step 1, 15 min at 250 V; step 2, 3 h voltage gradient to 10,000 V (linear ramp); step 3, 10,000 V until achieving 60,000 V/h (linear ramp). Second-dimension sodium dodecyl sulfate-polyacrylamide gel electrophoresis (SDS-PAGE) was performed in a Hoefer SE 900 vertical slab electrophoresis system (Hoefer, Holliston, MA, USA), with 12% (*w*/*v*) acrylamide gels, at 480 mA and 20 °C. After electrophoresis run the gels were stained with colloidal Coomassie blue G-250 [111]. The 2DE protein profiles were analyzed by gel scanning with a GS-800 calibrated densitometer (Bio-Rad) and the D analysis software (Bio-Rad) as described previously [49]. Protein spots detected by this procedure were excised from the gels for subsequent identification.

### 3.3. MALDI-TOF MS Analysis

Matrix-assisted laser desorption/ionization time-of-flight (MALDI-TOF/TOF) mass spectrometry (MS) measurements were performed to identify protein spots from 2DE gels. Protein spots were washed, distained, reduced, alkylated, and digested with trypsin following the procedure described by Osório and Reis [112]. The solution containing the peptides was collected and stored at −20 °C until application to a MALDI plate. Peptides were acidified with trifluoroacetic acid (TFA) and concentrated using C18 micro-columns (C18 Tips, 10 µL, Thermo Scientific, 87782). Peptides were thereafter eluted from the micro-column directly onto the MALDI plate with 1.5 µL of α-CHCA matrix (8 mg/mL) prepared in acetonitrile (50%, *v*/*v*), TFA (0.1%, *v*/*v*) and 6 mM ammonium phosphate. MALDI mass spectra were externally calibrated following the manufacturer’s instructions (TOF/TOF calibration mixture, AB SCIEX) and internal calibration was applied using trypsin autolysis peaks. Peptide mass spectra data was collected in positive ion reflector mode in the range of *m*/*z* 700–4000 (4800 Plus MALDI TOF/TOF Analyzer, AB SCIEX).

Proteins were identified by combining Peptide Mass Fingerprint and MS/MS information. Proteins were searched in a locally stored NCBI copy of protein sequences of the genomes of the sea anemones *Exaiptasia pallida* (26,042 protein count, GenBank accession: GCA_001417965.1) and *Nematostella vectensis* (24,780 protein count, GenBank accession: GCA_000209225.1), using the Mascot search engine (Version 2.4). The search included peaks with a signal-to-noise ratio greater than 10 and allowed for up to two missed trypsin cleavage sites, mass tolerance of 50 ppm, cysteine carbamidomethylation (fixed modification), methionine oxidation (variable modification), and a charge state of +1. For a match to be considered significant, protein scores with a probability greater than 95% (*p* < 0.05), calculated by the Mascot software, were required [112]. The data generated from 2D-MALDI procedures were also searched against UniProtKB protein sequence database in the Metazoa section [113,114], using the same parameters mentioned before.

### 3.4. In Solution Protein Digestion and MS/MS Analysis

For LC-MS/MS analysis, SF and IF protein samples were processed by filter aided sample preparation (FASP) method [115] with the following modifications. Protein samples (40 μg) were alkylated and digested with trypsin (recombinant, proteomics grade, Roche, Basel, Switzerland), at enzyme to protein ratio of 1:100 (*w*/*w*), for 16 h at 37 °C, in centrifugal filter units with nominal molecular weight limit (NMWL) of 30 kDa (MRCF0R030, Millipore, Billerica, MA, USA). Peptides were subsequently recovered by centrifugal filtration, acidified with formic acid (FA) (10%, *v*/*v*), desalted and concentrated by reversed-phase extraction (C18 Tips, 100 µL, Thermo Scientific, 87784) using acetonitrile (ACN) (70%, *v*/*v*) and TFA (0.1%, *v*/*v*) for peptide elution. Before LC–MS/MS, the peptides were recovered in 0.1% (*v*/*v*) Formic acid (FA) to the concentration of 0.04–0.06 μg/μL.

FASP protein digests (duplicate samples) were analyzed by nano-LC coupled to a hybrid Ion-trap mass spectrometer (LTQ Orbitrap Velos Pro—ETD, Thermo Scientific) as described previously [68]. Peptides were separated by reverse-phase chromatography (20 mm × 100 µm C18 precolumn followed by a 100 mm × 75 µm C18 column with particle size 5 µm, NanoSeparations, Nieuwkoop, The Netherlands) using a linear ascending gradient of buffer B (ACN + FA, 0.1%, *v*/*v*), being buffer A TFA, 0.1%, *v*/*v* in water. The gradient started from 2% B to 30% B in 40 min and to 95% B (*v*/*v*) in 30 min, at a flow rate of 0.3 µL/min (total elution time 70 min). Peptides were analyzed by on-line nano-electrospray ionization (easy nano-ESI) in positive mode, with Xcalibur software (version 2.6, Thermo Scientific). Full scans were performed at a resolution of 30,000 with scan ranges of 380–2000 *m*/*z*. The top 20 most intense ions were isolated and fragmented with CID by applying normalized collision energy of 30% value, isolation width of 2.0, activation time of 10 milliseconds and Q-value of 0.25. In total 4 nano-LC-MS/MS runs were performed.

### 3.5. Peptide Identification

The resulting ion-trap raw data (LTQ Orbitrap) were searched against custom cnidarians protein databases using MaxQuant freeware software (version 1.5.5.1) with the Andromeda search engine. MS and MS/MS tolerances were set to 10 ppm and 0.6 Da, respectively. Trypsin was selected for protein cleavage allowing for one missed cleavage. Carbamidomethylation and oxidation were selected as static and dynamic modifications, respectively. Identifications were validated by performing a decoy database search for the estimation of False Discovery Rate (FDR) and peptide identifications were accepted if they could be established at a probability greater than 95.0%. Protein identifications were accepted if they could be established at a probability greater than 99.9% and contained at least two identified peptides (Razor + unique peptides) [116,117], based on Occam’s razor principle). The protein database utilized was the locally stored NCBI copy of protein sequences of the genomes of the sea anemones *E. pallida* (26,042 protein count, GenBank accession: GCA_001417965.1), *N. vectensis* (24,780 protein count, GenBank accession: GCA_000209225.1), *Hydra vulgaris* (21,993 protein count, GenBank accession: GCF_000004095.1) and *Acropora digitifera* (33,878 protein count, GenBank accession: GCF_000222465.1). The identification of potential toxins was done against the manually reviewed venom proteins and toxins database, from the animal toxin annotation project of the UniProtKB/Swiss-Prot protein knowledgebase [118,119,120] (database size 1.20 MB, downloaded on 16 June 2016).

### 3.6. Protein Homology Search and GO Analysis

Protein sequences with unknown function were annotated with a blast search in the National Centre for Biotechnology Information database (NCBI, http://www.ncbi.nlm.nih.gov/) using blastp algorithm employing a threshold e-value of 1 × 10^−10^. Total of proteins identified with Maxquant software, were also blasted and mapped using the Blast2Go software (version 2.4.4) [73]. Gene ontology (GO) terms were used to group proteins within the domains of BP, CC, andMF.

## 4. Conclusions

The present work revealed for the first time a draft of the whole proteome of the sea anemone *B. verrucosa*. The shotgun proteomics analysis yielded most of the protein identified in a total of 412, whereas gel-based analyses provided less data but useful as complementary information. Altogether, both gel-based and gel-free approaches of proteomics analyses and functional bioinformatics analyses revealed three major groups of proteins belonging to “metabolic process”, “binding” and “cell parts” GO categories. Unlike throughput analyses, only eight proteins were identified from two-dimensional electrophoresis combined with MALDI-TOF/TOF. These eight proteins comprised enzymes mainly involved in the glycolytic pathway, antioxidants activities and RNA degradation. Notably, according to the results of KEGG analysis a significant number of enzymes corresponded to the Biosynthesis of antibiotics pathway indicating the importance of the biological antimicrobial chemical defense mechanisms. Moreover, some potential toxins such as metalloproteinases, and neurotoxin such as SE-cephalotoxin were identified. The combination of proteomic evidences and the ecology of the species, shed light about its strategy to subdue preys like mussels. In this sense, the toxins seemingly act synergically. Metalloproteinase may produce a degradation of the tissues, aiding the diffusion of the neurotoxins to the target, producing muscle paralysis. Hence, this work constitutes a reference proteome for future studies in sea anemones, also given insight about its potential toxin production and its putative mechanism of action in feeding.

## Figures and Tables

**Table 1:** Blast Search summary. Information concerning the proteins identification by Matrix-assisted laser desorption/ionization time-of-flight (MALDI-TOF/TOF) mass spectrometry of the proteins separated in two-dimensional gel electrophoresis.

Protein Name ^1^	Species ^2^	Protein ^3^ Score	Accession ^4^ Number	Ion ^5^ Score	Peptide Sequence ^6^
predicted protein (Peroxiredoxin)	*Nematostella vectensis*	137	XP_001640260.1	15	R.LIQAFQFTDK.H
				115	K.DYGVLLEDQGVALR.G
Ferritin	*Nematostella vectensis*	124	XP_001632011.1	114	R.QNYHEECEAGINK.Q
Ferritin	*Nematostella vectensis*	117	XP_001627474.1	11	K.LMKFQNQR.G
				97	R.QNYHEECEAGINK.Q
predicted protein (Ribonuclease)	*Nematostella vectensis*	106	XP_001634183.1	93	R.VEIEAIAIVGEVKDE.
Superoxide dismutase [Mn]	*Exaiptasia pallida*	428	KXJ18609.1	76	K.DFGSFENFK.X
				67	K.KDFGSFENFK.
				103	K.AIYDVIDWTNVADR.Y
Triosephosphate isomerase	*Nematostella vectensis*	356	XP_001633516.1	56	K.FFVGGNWK.M
				22	R.KFFVGGNWK.M
				95	K.VIACIGELLSER.E
				19	R.NIFGEKDELIGEK.V
				121	K.VVIAYEPVWAIGTGK.T
predicted protein/Alpha-enolase	*Nematostella vectensis*	95	XP_001632906.1	10	K.YNQLLR.I
				37	R.AAVPSGASTGIYEALELR.D
				10	K.LAMQEFMLLPTGASNFR.E
Fructose-bisphosphate aldolase	*Nematostella vectensis*	151	XP_001629735.1	41	K.LTFSFGR.A
				23	R.LLRDQGIIPGIK.V
				28	R.LANIGVENTEENRR.L
				24	R.LLRDQGIIPGIKVDK.G
Fructose-bisphosphate aldolase	*Nematostella vectensis*	97	XP_001629735.1	28	K.LTFSFGR.A
				32	R.LANIGVENTEENRR.L

^1^ best hit NCBI accession number; ^2^ the name of the species best hit belongs; ^3^ Score obtained for the MS ion; ^4^ NCBI accession number retrieved from the custom database; ^5^ MASCOT’s score for ion peptides; ^6^ peptides sequences identified with statistical significance.

**Table 2:** Top twenty Kyoto Encyclopedia of Genes and Genomes (KEGG) pathways.

Pathway	#Proteins in the Pathway	#Enzymes in Pathway
Purine metabolism	18	3
Thiamine metabolism	17	1
Biosynthesis of antibiotics	14	13
Glycolysis/Gluconeogenesis	9	6
Carbon fixation in photosynthetic organisms	9	6
Amino sugar and nucleotide sugar metabolism	6	3
Methane metabolism	6	3
Pyruvate metabolism	5	4
Cysteine and methionine metabolism	4	5
Citrate cycle (TCA cycle)	4	3
Fructose and mannose metabolism	4	2
Various types of *N*-glycan biosynthesis	4	1
Glycosphingolipid biosynthesis—ganglio series	4	1
Glycosaminoglycan degradation	4	1
Glycosphingolipid biosynthesis—globo and isoglobo series	4	1
Other glycan degradation	4	1
Glyoxylate and dicarboxylate metabolism	3	3
Carbon fixation pathways in prokaryotes	3	2
Pentose phosphate pathway	3	2
Histidine metabolism	2	2

**Table 3:** Potential toxins from the sea anemone *Bunodactis verrucosa*. Potential toxins identified by MaxQuant software against the venom section of UniProtKB/Swiss-Prot database.

Protein ^1^ Name	Species ^2^	Score ^3^	Accession ^4^ Number	Ion ^5^ Score	Peptide Sequence ^6^	Fraction ^7^ (Rep.)
SE-cephalotoxin	*Sepia esculenta*	11.47	CTX_SEPES	62.7	AGYIMGNR	IF (1)
				42.8	LDQINDKLDK	IF (1)
Basic phospholipase A2 vurtoxin	*Vipera renardi*	12.06	PA2B_VIPRE	2.9	CCFVHDCCYGNLPDCNPKIDR	SF (1)
				18.3	NGAIVCGK	IF (1)
Alpha-latroinsectotoxin-Lt1a	*Latrodectus tredecimguttatus*	11.73	LITA_LATTR	22.7	EMGRKLDK	IF (2)
				3.01	NSCMHNDKGCCFPWSCVCWSQTVSR	SF (1)
Zinc metalloproteinase/disintegrin	*Deinagkistrodon acutus*	11.48	VM2M2_DEIAC	27.4	FPYQGSSIILESGNVNDYEVVYPRK	SF (1)
				31.7	NTLESFGEWRAR	IF (1)
Neprilysin-1	*Trittame loki*	11.49	NEP_TRILK	28.4	LAHETNPR	IF (1)
				71.3	LEAMINK	SF (2)

^1^ UniProtKB/Swiss-Prot name of the protein identified as potential toxin; ^2^ name of the species best hit belongs; ^3^ Protein score which is derived from peptide posterior error probabilities; ^4^ UniProtKB/Swiss-Prot hit accession number; ^5^ Andromeda score for the best associated MS/MS spectrum; ^6^ UniProtKB/Swiss-Prot accession number; ^7^ fraction (IF: Insoluble fraction; SF: Soluble fraction) where a peptide was detected and replicates they occurred.

## Supplementary Materials

The following are available online at http://www.mdpi.com/1660-3397/16/2/42/s1, Figure S1: Two-dimensional gel electrophoresis of insoluble fraction (IF) from *Bunodactis verrucosa*, Figure S2: Combined Graph obtained for GO Distribution by Level (2); Table S1: Proteins identified against custom cnidarians databases title; Table S2: Proteins identified as potential toxins; Table S3: Details of GO annotation and protein accession number obtained with the Balst2Go software; Table S4: Details of the KEGG analyses obtained with the Balst2Go software.

## References

[1] Yan L., Herrington J., Goldberg E., Dulski P.M., Bugianesi R.M., Slaughter R.S., Banerjee P., Brochu R.M., Priest B.T., Kaczorowski G.J. (2005). *Stichodactyla helianthus* peptide, a pharmacological tool for studying Kv3.2 channels. Mol. Pharmacol..

[2] Tejuca M., Diaz I., Figueredo R., Roque L., Pazos F., Martinez D., Iznaga-Escobar N., Perez R., Alvarez C., Lanio M.E. (2004). Construction of an immunotoxin with the pore forming protein sti and ior C5, a monoclonal antibody against a colon cancer cell line. Int. Immunopharmacol..

[3] Beeton C., Pennington M.W., Wulff H., Singh S., Nugent D., Crossley G., Khaytin I., Calabresi P.A., Chen C.Y., Gutman G.A. (2005). Targeting effector memory t cells with a selective peptide inhibitor of Kv1.3 channels for therapy of autoimmune diseases. Mol. Pharmacol..

[4] Chi V., Pennington M.W., Norton R.S., Tarcha E.J., Londono L.M., Sims-Fahey B., Upadhyay S.K., Lakey J.T., Iadonato S., Wulff H. (2012). Development of a sea anemone toxin as an immunomodulator for therapy of autoimmune diseases. Toxicon.

[5] Turk T., Kem W.R. (2009). The phylum cnidaria and investigations of its toxins and venoms until 1990. Toxicon.

[6] WoRMS World Register of Marine Species. http://www.marinespecies.org/aphia.php?p=taxdetails&id=1267.

[7] Daly M., Brugler M.R., Cartwright P., Collins A.G., Dawson M.N., Fautin D.G., France S.C., McFadden C.S., Opresko D.M., Rodriguez E. (2007). The phylum cnidaria: A review of phylogenetic patterns and diversity 300 years after Linnaeus. Zootaxa.

[8] Fautin D.G. (2009). Structural diversity, systematics, and evolution of cnidae. Toxicon.

[9] Frazão B., Vasconcelos V., Antunes A. (2012). Sea anemone (Cnidaria, Anthozoa, Actiniaria) toxins: An overview. Mar. Drugs.

[10] Beress L. (1982). Biologically active compounds from coelenterates. Pure Appl. Chem..

[11] Aneiros A., Garateix A. (2004). Bioactive peptides from marine sources: Pharmacological properties and isolation procedures. J. Chromatogr. B Anal. Technol. Biomed. Life Sci..

[12] Aneiros A., Garcia I., Martinez J.R., Harvey A.L., Anderson A.J., Marshall D.L., Engstrom A., Hellman U., Karlsson E. (1993). A potassium channel toxin from the secretion of the sea anemone *Bunodosoma granulifera*. Isolation, amino acid sequence and biological activity. Biochim. Biophys. Acta.

[13] Salinas E.M., Cebada J., Valdes A., Garateix A., Aneiros A., Alvarez J.L. (1997). Effects of a toxin from the mucus of the Caribbean sea anemone (*Bunodosoma granulifera*) on the ionic currents of single ventricular mammalian cardiomyocytes. Toxicon.

[14] Garateix A., Castellanos M., Hernandez J.L., Mas R., Menendez R., Romero L., Chavez M. (1992). Effects of a high molecular weight toxin from the sea anemone *Condylactis gigantea* on cholinergic responses. Comp. Biochem. Physiol. C.

[15] Garateix A., Flores A., Garcia-Andrade J.M., Palmero A., Aneiros A., Vega R., Soto E. (1996). Antagonism of glutamate receptors by a chromatographic fraction from the exudate of the sea anemone *Phyllactis flosculifera*. Toxicon.

[16] Garateix A., Salceda E., López O., Salazar H., Aneiros A., Zaharenko A.J., de Freitas J.C. (2006). Pharmacological characterization of *Bunodosoma* toxins on mammalian voltage dependt sodium channels. Pharmacologyonline.

[17] Garateix A., Vega R., Salceda E., Cebada J., Aneiros A., Soto E. (2000). Bgk anemone toxin inhibits outward K(+) currents in snail neurons. Brain Res..

[18] Castañeda O., Harvey A.L. (2009). Discovery and characterization of cnidarian peptide toxins that affect neuronal potassium ion channels. Toxicon.

[19] Castañeda O., Sotolongo V., Amor A.M., Stöcklin R., Anderson A.J., Harvey A.L., Engström Å., Wernstedt C., Karlsson E. (1995). Characterization of a potassium channel toxin from the caribbean sea anemone *Stichodactyla helianthus*. Toxicon.

[20] Cotton J., Crest M., Bouet F., Alessandri N., Gola M., Forest E., Karlsson E., Castaneda O., Harvey A.L., Vita C. (1997). A potassium-channel toxin from the sea anemone *Bunodosoma granulifera*, an inhibitor for Kv1 channels. Revision of the amino acid sequence, disulfide-bridge assignment, chemical synthesis, and biological activity. Eur. J. Biochem..

[21] Standker L., Beress L., Garateix A., Christ T., Ravens U., Salceda E., Soto E., John H., Forssmann W.G., Aneiros A. (2006). A new toxin from the sea anemone *Condylactis gigantea* with effect on sodium channel inactivation. Toxicon.

[22] Rodríguez A.A., Cassoli J.S., Sa F., Dong Z.Q., de Freitas J.C., Pimenta A.M.C., de Lima M.E., Konno K., Lee S.M.Y., Garateix A. (2012). Peptide fingerprinting of the neurotoxic fractions isolated from the secretions of sea anemones *Stichodactyla helianthus* and *Bunodosoma granulifera*. New members of the APETx-like family identified by a 454 pyrosequencing approach. Peptides.

[23] Rodriguez A.A., Salceda E., Garateix A.G., Zaharenko A.J., Peigneur S., Lopez O., Pons T., Richardson M., Diaz M., Hernandez Y. (2014). A novel sea anemone peptide that inhibits acid-sensing ion channels. Peptides.

[24] Lanio M.E., Morera V., Alvarez C., Tejuca M., Gomez T., Pazos F., Besada V., Martinez D., Huerta V., Padron G. (2001). Purification and characterization of two hemolysins from *Stichodactyla helianthus*. Toxicon.

[25] Rodriguez A.A., Standker L., Zaharenko A.J., Garateix A.G., Forssmann W.G., Beress L., Valdes O., Hernandez Y., Laguna A. (2012). Combining multidimensional liquid chromatography and MALDI-TOF-MS for the fingerprint analysis of secreted peptides from the unexplored sea anemone species *Phymanthus crucifer*. J. Chromatogr. B Anal. Technol. Biomed. Life Sci..

[26] Lanio M.E., Alvarez C., Ochoa C., Ros U., Pazos F., Martinez D., Tejuca M., Eugenio L.M., Casallanovo F., Dyszy F.H. (2007). Sticholysins I and II interaction with cationic micelles promotes toxins’ conformational changes and enhanced hemolytic activity. Toxicon.

[27] Alvarez C., Casallanovo F., Shida C.S., Nogueira L.V., Martinez D., Tejuca M., Pazos I.F., Lanio M.E., Menestrina G., Lissi E. (2003). Binding of sea anemone pore-forming toxins Sticholysins I and II to interfaces—Modulation of conformation and activity, and lipid-protein interaction. Chem. Phys. Lipids.

[28] Álvarez C., Mancheño J.M., Martínez D., Tejuca M., Pazos F., Lanio M.E. (2009). Sticholysins, two pore-forming toxins produced by the Caribbean sea anemone *Stichodactyla helianthus*: Their interaction with membranes. Toxicon.

[29] Moran Y., Gordon D., Gurevitz M. (2009). Sea anemone toxins affecting voltage-gated sodium channels—Molecular and evolutionary features. Toxicon.

[30] Moran Y., Weinberger H., Sullivan J.C., Reitzel A.M., Finnerty J.R., Gurevitz M. (2008). Concerted evolution of sea anemone neurotoxin genes is revealed through analysis of the *Nematostella vectensis* genome. Mol. Biol. Evol..

[31] Nesher N., Shapira E., Sher D., Moran Y., Tsveyer L., Turchetti-Maia A.L., Horowitz M., Hochner B., Zlotkin E. (2013). Ade-1, a new inotropic Na(+) channel toxin from *Aiptasia diaphana*, is similar to, yet distinct from, known anemone Na(+) channel toxins. Biochem. J..

[32] Orts D.J., Moran Y., Cologna C.T., Peigneur S., Madio B., Praher D., Quinton L., De Pauw E., Bicudo J.E., Tytgat J. (2013). Bcstx3 is a founder of a novel sea anemone toxin family of potassium channel blocker. FEBS J..

[33] Hasegawa Y., Honma T., Nagai H., Ishida M., Nagashima Y., Shiomi K. (2006). Isolation and cDNA cloning of a potassium channel peptide toxin from the sea anemone *Anemonia erythraea*. Toxicon.

[34] Honma T., Kawahata S., Ishida M., Nagai H., Nagashima Y., Shiomi K. (2008). Novel peptide toxins from sea anemone *Stichodactyla haddoni*. Peptides.

[35] Honma T., Shiomi K. (2006). Peptide toxins in sea anemones: Structural and functional aspects. Mar. Biotechnol..

[36] Minagawa S., Ishida M., Nagashima Y., Shiomi K. (1998). Primary structure of a potassium channel toxin from the sea anemone *Actinia equina*. FEBS Lett..

[37] Oliveira J.S., Fuentes-Silva D., King G.F. (2012). Development of a rational nomenclature for naming peptide and protein toxins from sea anemones. Toxicon.

[38] Urbarova I., Karlsen B.O., Okkenhaug S., Seternes O.M., Johansen S.D., Emblem Å. (2012). Digital marine bioprospecting: Mining new neurotoxin drug candidates from the transcriptomes of cold-water sea anemones. Mar. Drugs.

[39] Macrander J., Brugler M.R., Daly M. (2015). A RNA-seq approach to identify putative toxins from acrorhagi in aggressive and non-aggressive *Anthopleura elegantissima* polyps. BMC Genom..

[40] Oliveira J.S., Fuentes-Silva D., Zaharenko A.J., de Lima M.E., Pimenta A.M., Martin-Eauclaire M.F., Zingali R.B., Rochat H. (2009). Sea anemone peptides. Biological activities, structure-function relationships and phylogenetic aspects. Animal Toxins: State of the Art. Perspective in Health and Biotechnology.

[41] Norton R.S. (1991). Structure and structure-function relationships of sea anemone proteins that interact with the sodium channel. Toxicon.

[42] Norton R.S. (2009). Structures of sea anemone toxins. Toxicon.

[43] Fautin D.G., Malarky L., Soberón J. (2013). Latitudinal diversity of sea anemones (Cnidaria: Actiniaria). Biol. Bull..

[44] Frazão B. (2017). A Genomic and Proteomic Study of Sea Anemones and Jellyfish from Portugal. Ph.D. Thesis.

[45] Fautin D. Bunodactis verrucosa. In: Hexacorallians (Actiniaria) of the World. Accessed through: World Register of Marine Species.

[46] Frazão B., Antunes A. (2016). Jellyfish bioactive compounds: Methods for wet-lab work. Mar. Drugs.

[47] Putnam N.H., Srivastava M., Hellsten U., Dirks B., Chapman J., Salamov A., Terry A., Shapiro H., Lindquist E., Kapitonov V.V. (2007). Sea anemone genome reveals ancestral eumetazoan gene repertoire and genomic organization. Science.

[48] Zhuang Y., Hou H., Zhao X., Zhang Z., Li B. (2009). Effects of collagen and collagen hydrolysate from jellyfish (*Rhopilema esculentum*) on mice skin photoaging induced by uv irradiation. J. Food Sci..

[49] Campos A., Puerto M., Prieto A., Cameán A., Almeida A.M., Coelho A.V., Vasconcelos V. (2013). Protein extraction and two-dimensional gel electrophoresis of proteins in the marine mussel *Mytilus galloprovincialis*: An important tool for protein expression studies, food quality and safety assessment. J. Sci. Food Agric..

[50] Puerto M., Campos A., Prieto A., Cameán A., de Almeida A.M., Coelho A.V., Vasconcelos V. (2011). Differential protein expression in two bivalve species; *Mytilus galloprovincialis* and *Corbicula fluminea*; exposed to *Cylindrospermopsis raciborskii* cells. Aquat. Toxicol..

[51] Frazão B., Campos A., Osório H., Thomas B., Leandro S., Teixeira A., Vasconcelos V., Antunes A. (2017). Analysis of *Pelagia noctiluca* proteome reveals a red fluorescent protein, a zinc metalloproteinase and a peroxiredoxin. Protein J..

[52] Zhang X. (2015). Less is more: Membrane protein digestion beyond urea–trypsin solution for next-level proteomics. Mol. Cell. Proteom..

[53] Wu C.C., MacCoss M.J., Howell K.E., Yates J.R. (2003). A method for the comprehensive proteomic analysis of membrane proteins. Nat. Biotechnol..

[54] Ji H., Wang J., Guo J., Li Y., Lian S., Guo W., Yang H., Kong F., Zhen L., Guo L. (2016). Progress in the biological function of alpha-enolase. Anim. Nutr..

[55] Iida H., Yahara I. (1985). Yeast heat-shock protein of *M*r 48,000 is an isoprotein of enolase. Nature.

[56] Butterworth P. (1998). Book review: Ribonucleases: Structure and function. G. D’Alessio and J. F. Riordan (Eds.). Academic press, new york and london. 670 + xix pages (1997). Cell Biochem. Funct..

[57] Davenport R.C., Bash P.A., Seaton B.A., Karplus M., Petsko G.A., Ringe D. (1991). Structure of the triosephosphate isomerase-phosphoglycolohydroxamate complex: An analog of the intermediate on the reaction pathway. Biochemistry.

[58] Cooper S.J., Leonard G.A., McSweeney S.M., Thompson A.W., Naismith J.H., Qamar S., Plater A., Berry A., Hunter W.N. (1996). The crystal structure of a class II fructose-1, 6-bisphosphate aldolase shows a novel binuclear metal-binding active site embedded in a familiar fold. Structure.

[59] Horecker B., Tsolas O., Lai C. (1972). 6 aldolases. Enzymes.

[60] Harrison P.M., Arosio P. (1996). The ferritins: Molecular properties, iron storage function and cellular regulation. Biochim. Biophys. Acta Bioenerg..

[61] Arosio P., Levi S. (2002). Ferritin, iron homeostasis, and oxidative damage. Free Radic. Biol. Med..

[62] Kim K., Kim I., Lee K.-Y., Rhee S., Stadtman E. (1988). The isolation and purification of a specific “protector” protein which inhibits enzyme inactivation by a thiol/Fe (III)/O2 mixed-function oxidation system. J. Biol. Chem..

[63] Rhee S.G., Woo H.A., Kil I.S., Bae S.H. (2012). Peroxiredoxin functions as a peroxidase and a regulator and sensor of local peroxides. J. Biol. Chem..

[64] Cui H., Wang Y., Wang Y., Qin S. (2012). Genome-wide analysis of putative peroxiredoxin in unicellular and filamentous cyanobacteria. BMC Evol. Biol..

[65] Maksimenko A.V., Vavaev A.V. (2012). Antioxidant enzymes as potential targets in cardioprotection and treatment of cardiovascular diseases. Enzyme antioxidants: The next stage of pharmacological counterwork to the oxidative stress. Heart Int..

[66] Ruan Z., Liu G., Wang B., Zhou Y., Lu J., Wang Q., Zhao J., Zhang L. (2014). First report of a peroxiredoxin homologue in jellyfish: Molecular cloning, expression and functional characterization of CcPrx4 from *Cyanea capillata*. Mar. Drugs.

[67] Fukai T., Ushio-Fukai M. (2011). Superoxide dismutases: Role in redox signaling, vascular function, and diseases. Antioxid. Redox Signal..

[68] Campos A., Apraiz I., da Fonseca R.R., Cristobal S. (2015). Shotgun analysis of the marine mussel *Mytilus edulis* hemolymph proteome and mapping the innate immunity elements. Proteomics.

[69] Campos A., Danielsson G., Farinha A.P., Kuruvilla J., Warholm P., Cristobal S. (2016). Shotgun proteomics to unravel marine mussel (*Mytilus edulis*) response to long-term exposure to low salinity and propranolol in a baltic sea microcosm. J. Proteom..

[70] Culma M.F., Pereanez J.A., Rangel V.N., Lomonte B. (2014). Snake venomics of *Bothrops punctatus*, a semiarboreal pitviper species from Antioquia, Colombia. PeerJ.

[71] Zhang Y., Fonslow B.R., Shan B., Baek M.-C., Yates III J.R. (2013). Protein analysis by shotgun/bottom-up proteomics. Chem. Rev..

[72] Keller B.O., Sui J., Young A.B., Whittal R.M. (2008). Interferences and contaminants encountered in modern mass spectrometry. Anal. Chim. Acta.

[73] Conesa A., Gotz S., Garcia-Gomez J., Terol J., Talon M., Robles M. (2005). Blast2go: A universal tool for annotation, visualization and analysis in functional genomics research. Bioinformatics.

[74] Daly M., Gusmão L.C., Reft A.J., Rodríguez E. (2010). Phylogenetic signal in mitochondrial and nuclear markers in sea anemones (Cnidaria, Actiniaria). Integr. Comp. Biol..

[75] Moffatt B.A., Ashihara H. (2002). Purine and pyrimidine nucleotide synthesis and metabolism. Arabidopsis Book.

[76] Lonsdale D. (2006). A review of the biochemistry, metabolism and clinical benefits of thiamin (e) and its derivatives. Evid. Based Complement. Altern. Med..

[77] Borbón H., Váldes S., Alvarado-Mesén J., Soto R., Vega I. (2016). Antimicrobial properties of sea anemone anthopleura nigrescens from pacific coast of costa rica. Asian Pac. J. Trop. Biomed..

[78] Rocha J., Peixe L., Gomes N., Calado R. (2011). Cnidarians as a source of new marine bioactive compounds—An overview of the last decade and future steps for bioprospecting. Mar. Drugs.

[79] Mariottini G.L., Grice I.D. (2016). Antimicrobials from cnidarians. A new perspective for anti-infective therapy?. Mar. Drugs.

[80] Davy S.K., Allemand D., Weis V.M. (2012). Cell biology of cnidarian-dinoflagellate symbiosis. Microb. Mol. Biol. Rev..

[81] Bellis E.S., Howe D.K., Denver D.R. (2016). Genome-wide polymorphism and signatures of selection in the symbiotic sea anemone *Aiptasia*. BMC Genom..

[82] Tsai I.H., Wang Y.M., Chiang T.Y., Chen Y.L., Huang R.J. (2000). Purification, cloning and sequence analyses for pro-metalloprotease-disintegrin variants from *Deinagkistrodon acutus* venom and subclassification of the small venom metalloproteases. Eur. J. Biochem..

[83] Undheim E.A., Sunagar K., Herzig V., Kely L., Low D.H., Jackson T.N., Jones A., Kurniawan N., King G.F., Ali S.A. (2013). A proteomics and transcriptomics investigation of the venom from the barychelid spider *Trittame loki* (brush-foot trapdoor). Toxins.

[84] Sarras M.P., Li Y., Leontovich A., Zhang J.S. (2002). Structure, expression, and developmental function of early divergent forms of metalloproteinases in hydra. Cell Res..

[85] Leontovich A.A., Zhang J., Shimokawa K.-I., Nagase H., Sarras M. (2000). A novel hydra matrix metalloproteinase (hmmp) functions in extracellular matrix degradation, morphogenesis and the maintenance of differentiated cells in the foot process. Development.

[86] Yan L., Fei K., Zhang J., Dexter S., Sarras M. (2000). Identification and characterization of hydra metalloproteinase 2 (HMP2): A meprin-like astacin metalloproteinase that functions in foot morphogenesis. Development.

[87] Pan T.-L., Gröger H., Schmid V., Spring J. (1998). A toxin homology domain in an astacin-like metalloproteinase of the jellyfish podocoryne carnea with a dual role in digestion and development. Dev. Genes Evol..

[88] Lee H., Jung E.-S., Kang C., Yoon W.D., Kim J.-S., Kim E. (2011). Scyphozoan jellyfish venom metalloproteinases and their role in the cytotoxicity. Toxicon.

[89] Moran Y., Praher D., Schlesinger A., Ayalon A., Tal Y., Technau U. (2013). Analysis of soluble protein contents from the nematocysts of a model sea anemone sheds light on venom evolution. Mar. Biotechnol..

[90] Tsai I.-H., Wang Y.-M., Cheng A.C., Starkov V., Osipov A., Nikitin I., Makarova Y., Ziganshin R., Utkin Y. (2011). cDNA cloning, structural, and functional analyses of venom phospholipases a 2 and a kunitz-type protease inhibitor from steppe viper *Vipera usinii renardi*. Toxicon.

[91] Fry B.G., Roelants K., Champagne D.E., Scheib H., Tyndall J.D., King G.F., Nevalainen T.J., Norman J.A., Lewis R.J., Norton R.S. (2009). The toxicogenomic multiverse: Convergent recruitment of proteins into animal venoms. Annu. Rev. Genom. Hum. Genet..

[92] Valentin E., Ghomashchi F., Gelb M.H., Lazdunski M., Lambeau G. (2000). Novel human secreted phospholipase A2 with homology to the group III bee venom enzyme. J. Biol. Chem..

[93] Fry B. (2015). Venomous Reptiles and Their Toxins: Evolution, Pathophysiology and Biodiscovery.

[94] Nevalainen T.J. (2008). Phospholipases a 2 in the genome of the sea anemone *Nematostella vectensis*. Comp. Biochem. Physiol. Part D Genom. Proteom..

[95] Fry B.G., Wüster W. (2004). Assembling an arsenal: Origin and evolution of the snake venom proteome inferred from phylogenetic analysis of toxin sequences. Mol. Biol. Evol..

[96] Komori Y., Nikai T., Sugihara H. (1988). Purification and characterization of a lethal toxin from the venom of *Heloderma horridum horridum*. Biochem. Biophys. Res. Commun..

[97] Fry B.G., Roelants K., Winter K., Hodgson W.C., Griesman L., Kwok H.F., Scanlon D., Karas J., Shaw C., Wong L. (2009). Novel venom proteins produced by differential domain-expression strategies in beaded lizards and Gila monsters (genus *Heloderma*). Mol. Biol. Evol..

[98] Jouiaei M., Yanagihara A.A., Madio B., Nevalainen T.J., Alewood P.F., Fry B.G. (2015). Ancient venom systems: A review on cnidaria toxins. Toxins.

[99] Skewes M., Tyler-Walters H., Hiscock K. (2007). Aulactinia verrucosa Gem Anemone.

[100] White B. (2004). Anthopleura xanthogrammica.

[101] Sebens K.P. (1981). The allometry of feeding, energetics, and body size in three sea anemone species. Biol. Bull..

[102] Hand C., Uhlinger K.R. (1992). The culture, sexual and asexual reproduction, and growth of the sea anemone *Nematostella vectensis*. Biol. Bull..

[103] Cope W.G., Bringolf R.B., Buchwalter D.B., Newton T.J., Ingersoll C.G., Wang N., Augspurger T., Dwyer F.J., Barnhart M.C., Neves R.J. (2008). Differential exposure, duration, and sensitivity of unionoidean bivalve life stages to environmental contaminants. J. N. Am. Benthol. Soc..

[104] Gilroy È.A., Gillis P.L., King L.E., Bendo N.A., Salerno J., Giacomin M., de Solla S.R. (2017). The effects of pharmaceuticals on a unionid mussel (*Lampsilis siliquoidea*): An examination of acute and chronic endpoints of toxicity across life stages. Environ. Toxicol. Chem..

[105] Bleam D.E., Couch K.J., Distler D.A. (1999). Key to the unionid mussels of kansas. Trans. Kans. Acad. Sci. (1903).

[106] Ghiretti F. (1959). Cephalotoxin: The crab-paralysing agent of the posterior salivary glands of cephalopods. Nature.

[107] McDonald N., Cottrell G. (1972). Purification and mode of action of toxin from *Eledone cirrosa*. Comp. Gen. Pharmacol..

[108] Songdahl J., Shapiro B. (1974). Purification and composition of a toxin from the posterior salivary gland of *Octopus dofleini*. Toxicon.

[109] Cariello L., Zanetti L. (1977). α- and β-cephalotoxin: Two paralysing proteins from posterior salivary glands of *Octopus vulgaris*. Comp. Biochem. Physiol. C Comp. Pharmacol..

[110] Bradford M.M. (1976). A rapid and sensitive method for the quantitation of microgram quantities of protein utilizing the principle of protein-dye binding. Anal. Biochem..

[111] Neuhoff V., Arold N., Taube D., Ehrhardt W. (1988). Improved staining of proteins in polyacrylamide gels including isoelectric focusing gels with clear background at nanogram sensitivity using Coomassie Brilliant Blue G-250 and R-250. Electrophoresis.

[112] Osório H., Reis C.A., Matthiesen R. (2013). Mass Spectrometry Methods for Studying Glycosylation in Cancer. Mass Spectrometry Data Analysis in Proteomics, Methods in Molecular Biology (Methods and Protocols).

[113] Apweiler R., Bairoch A., Wu C.H., Barker W.C., Boeckmann B., Ferro S., Gasteiger E., Huang H., Lopez R., Magrane M. (2004). Uniprot: The universal protein knowledgebase. Nucleic Acids Res..

[114] The UniProt Consortium (2017). Uniprot: The universal protein knowledgebase. Nucleic Acids Res..

[115] Wisniewski J.R., Zougman A., Nagaraj N., Mann M. (2009). Universal sample preparation method for proteome analysis. Nat. Methods.

[116] Cox J., Mann M. (2008). Maxquant enables high peptide identification rates, individualized ppb-range mass accuracies and proteome-wide protein quantification. Nat. Biotechnol..

[117] Cox J., Matic I., Hilger M., Nagaraj N., Selbach M., Olsen J.V., Mann M. (2009). A practical guide to the maxquant computational platform for silac-based quantitative proteomics. Nat. Protoc..

[118] Jungo F., Estreicher A., Bairoch A., Bougueleret L., Xenarios I. (2010). Animal toxins: How is complexity represented in databases?. Toxins.

[119] Jungo F., Bairoch A. (2005). Tox-Prot, the toxin protein annotation program of the Swiss-Prot protein knowledgebase. Toxicon.

[120] Jungo F., Bougueleret L., Xenarios I., Poux S. (2012). The Uniprotkb/Swiss-Prot Tox-Prot program: A central hub of integrated venom protein data. Toxicon.

