# Supplementary material for: Proteomic Analyses of the Unexplored Sea Anemone Bunodactis verrucosa
Source: Mar Drugs. 2018 Jan 24;16(2):42. doi: 10.3390/md16020042 (PMC5852470; doi:10.3390/md16020042)
Supplement: Supplementary file 1 [file marinedrugs-16-00042-s001.zip › Figuere_S1_&_caption.docx]

Supplementary Materials: Proteomic analyses of the unexplored sea anemone *Bunodactis verrucosa*

Dany Domínguez-Pérez ^1,2,+^, Alexandre Campos ^1,2,+^, Armando Alexei Rodríguez ^3^, Maria V Turkina ^4^, Tiago Ribeiro ^1,2^, Hugo Osorio ^5,6,7^, Vítor Vasconcelos ^1,2^ and Agostinho Antunes ^1,2,^*

^1^ CIIMAR/CIMAR, Interdisciplinary Centre of Marine and Environmental Research, University of Porto, Terminal de Cruzeiros do Porto de Leixões, Av. General Norton de Matos, s/n, Porto 4450-208, Portugal; [danydguezperez@gmail.com](mailto:danydguezperez@gmail.com) (D.D.-P.); [amoclclix@gmail.com](mailto:amoclclix@gmail.com) (A.C.); [tiago.amribeiro8@gmail.com](mailto:tiago.amribeiro8@gmail.com) (T.R.); [vmvascon@fc.up.pt](mailto:vmvascon@fc.up.pt) (V.V.)

^2^ Biology Department, Faculty of Sciences, University of Porto, Rua do Campo Alegre, s/n, Porto 4169-007, Portugal

^3^ Department of Experimental and Clinical Peptide Chemistry, Hanover Medical School (MHH), Feodor-Lynen-Straße 31, D-30625 Hannover, Germany 1; [aara259@gmail.com](mailto:aara259@gmail.com)

^4^ Division of Cell Biology, Department of Clinical and Experimental Medicine, Linköping University, SE-581 85, Linköping, Sweden; [maria.turkina@liu.se](mailto:maria.turkina@liu.se)

^5^ i3S ‐ Instituto de Investigação e Inovação em Saúde, Universidade do Porto, Rua Alfredo Allen, 208, 4200‐135 Porto, Portugal; [hosorio@ipatimup.pt](mailto:hosorio@ipatimup.pt)

^6^ Ipatimup, Institute of Molecular Pathology and Immunology of the University of Porto, Rua Júlio Amaral de Carvalho, 45, 4200‐135 Porto, Portugal

^7^ Department of Pathology and Oncology, Faculty of Medicine, University of Porto, Al. Prof. Hernâni Monteiro, 4200‐319 Porto, Portugal

^+^ These authors contributed equally to this work

***** Correspondence: aantunes@ciimar.up.pt; Tel.: +353‐22‐340‐1813

Academic Editor: name

Received: date; Accepted: date; Published: date


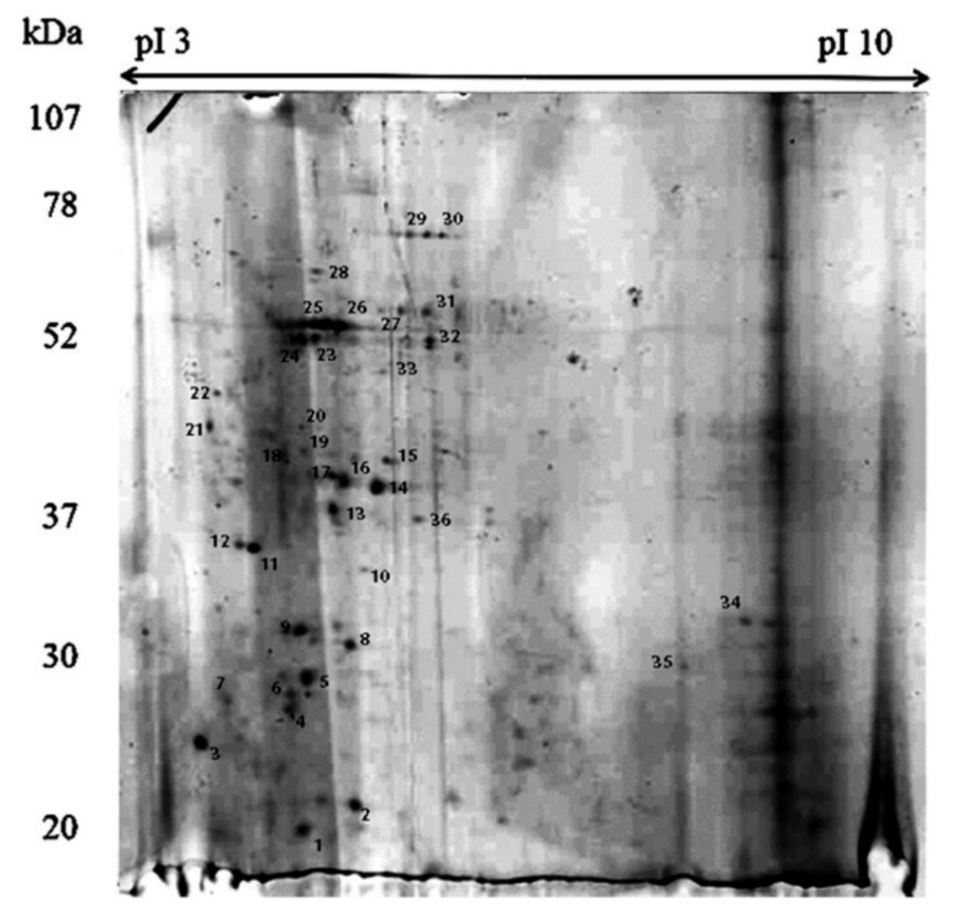


**Figure S1.** Two-dimensional gel electrophoresis of insoluble fraction (IF) from *Bunodactis verrucosa***.** The first dimension was carried out on 17 cm, pH 3-10 IEF gel strips and the second dimension on 12% SDS-PAGE gels. Gels were stained with colloidal Coomassie blue G-250. Spots selected for MALDI-TOF/TOF mass spectrometry analyses are indicated with numbers.

**Graph S1.** Combined Graph obtained for GO Distribution by Level (2). Details of GO annotation and protein accession number obtained by the Blast2Go software. The functional annotation based on the three major GO categories can be found as follow: Biological Process (BP), Molecular function (MF) and Cellular Process (CC).

**Table S1.** **Proteins identified against custom cnidarians databases.** Maxquant output file containing all the proteins retrieved from custom cnidarians databases. Could be found as a summary of the analysis and details of the group of protein identified with its corresponding peptide evidences. For better comprehension of the concepts employed in this work, was also attached a helpful file provided by MaxQuant team.

**Table S2.** **Proteins identified as potential toxins.** Maxquant output file containing the proteins identified as potential toxin retrieved from the manually reviewed venom proteins and toxins database, from the animal toxin annotation project of the UniProtKB/Swiss-Prot protein knowledgebase. Herein, was included a summary of the analysis and details of the group of protein identified with its corresponding peptide evidences.

**Table S3.** **Details of GO annotation and the corresponding accession number of the proteins obtained with the Balst2Go software.** Here is included the number of sequences (#Seqs) associated with each GO terms with its corresponding accession number, according to the three major categories: Biological Process (BP), Molecular Function (MF) and Cellular Component (CC). Plot of robust.

**Table S4.** **Details of the Kyoto Encyclopedia of Genes and Genomes (KEGG) analyses obtained with the Balst2Go software.** Table contains the number of enzyme involved in each pathway (#Enzs in Pathway), the number of sequences identified for each enzyme (#Seqs of Enzyme) and its corresponding accession number.
