# Supplementary figures and images for: Proteomic Analyses of the Unexplored Sea Anemone Bunodactis verrucosa
Source: Mar Drugs. 2018 Jan 24;16(2):42. doi: 10.3390/md16020042 (PMC5852470; doi:10.3390/md16020042)

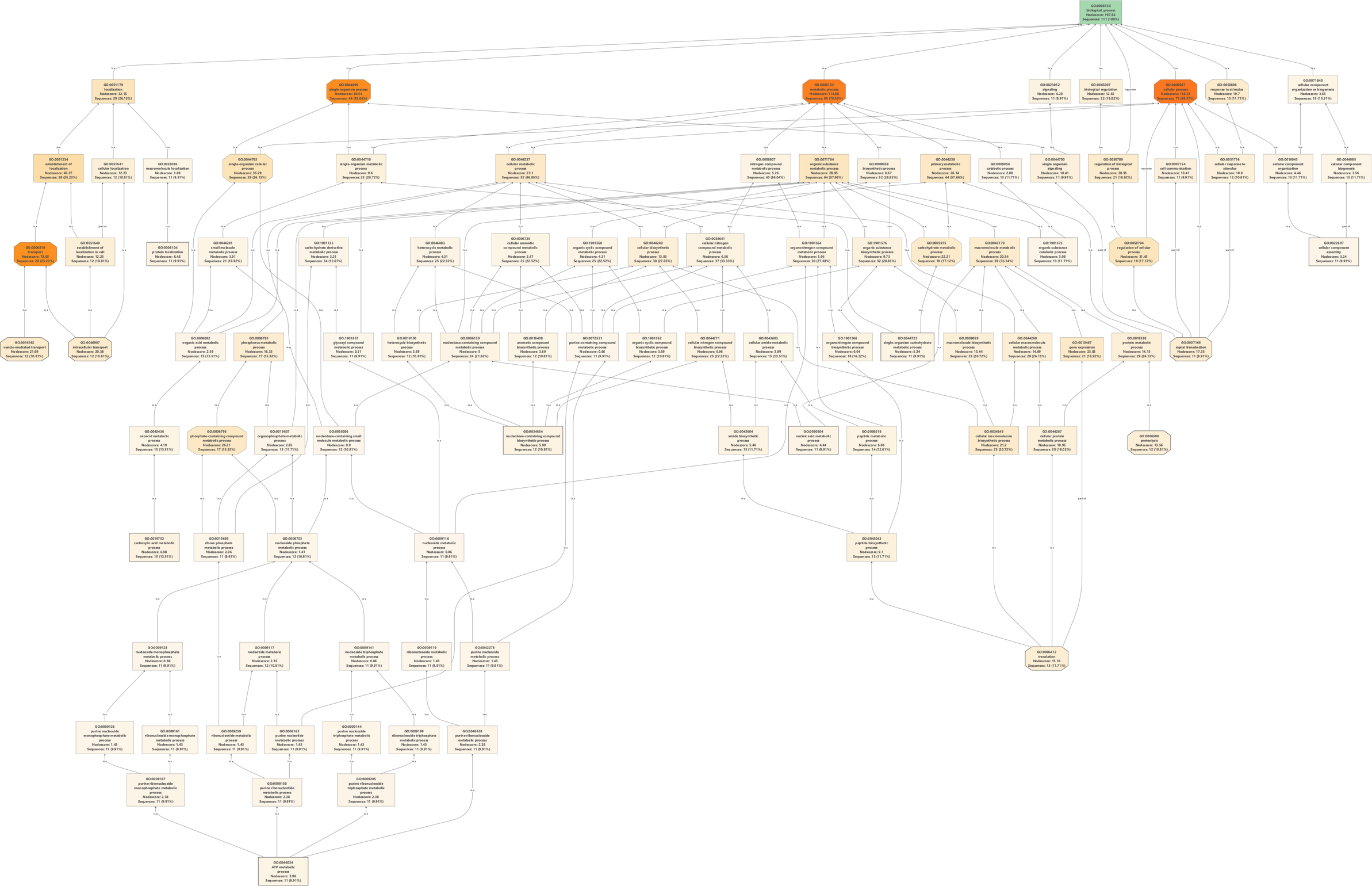

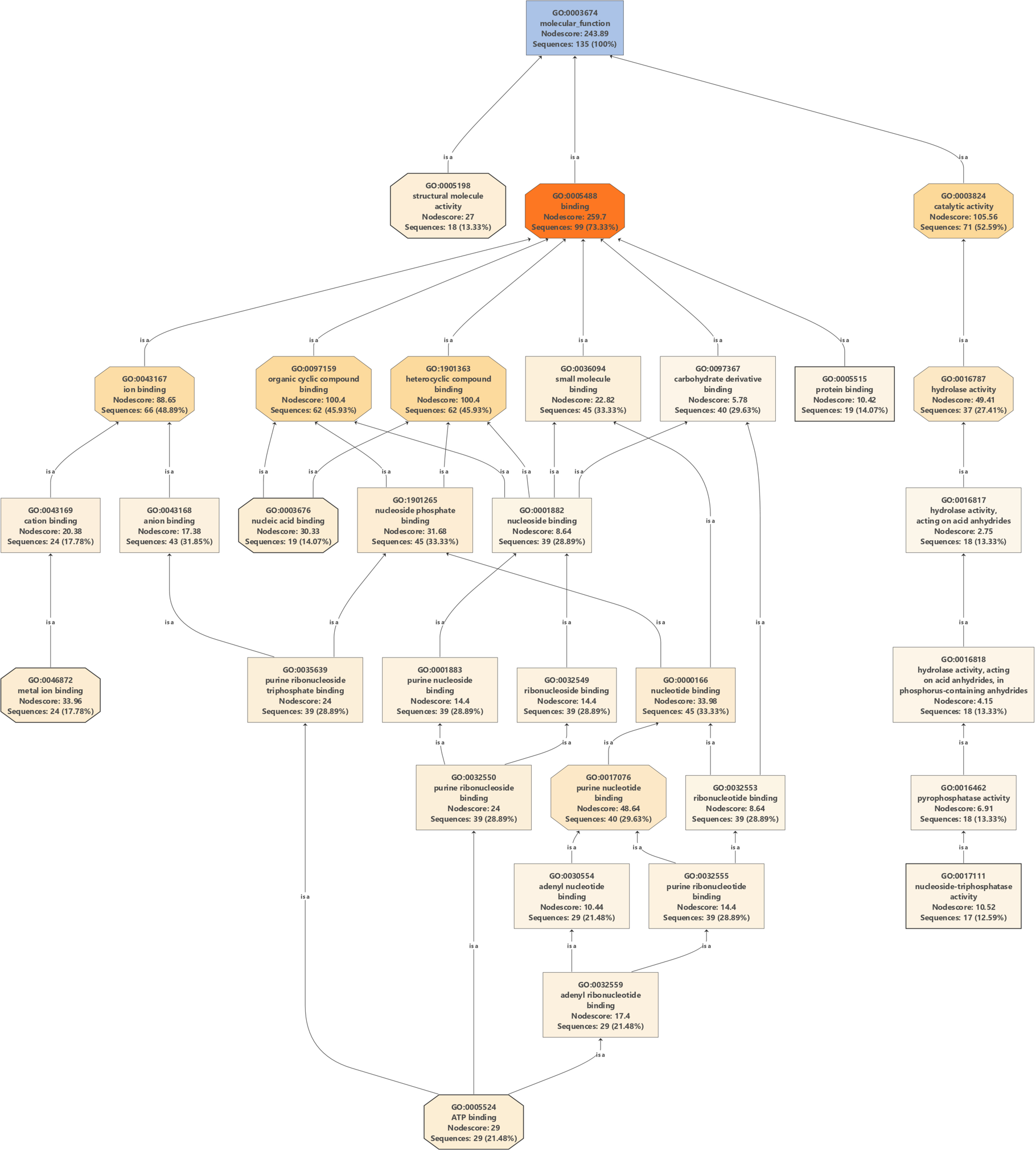

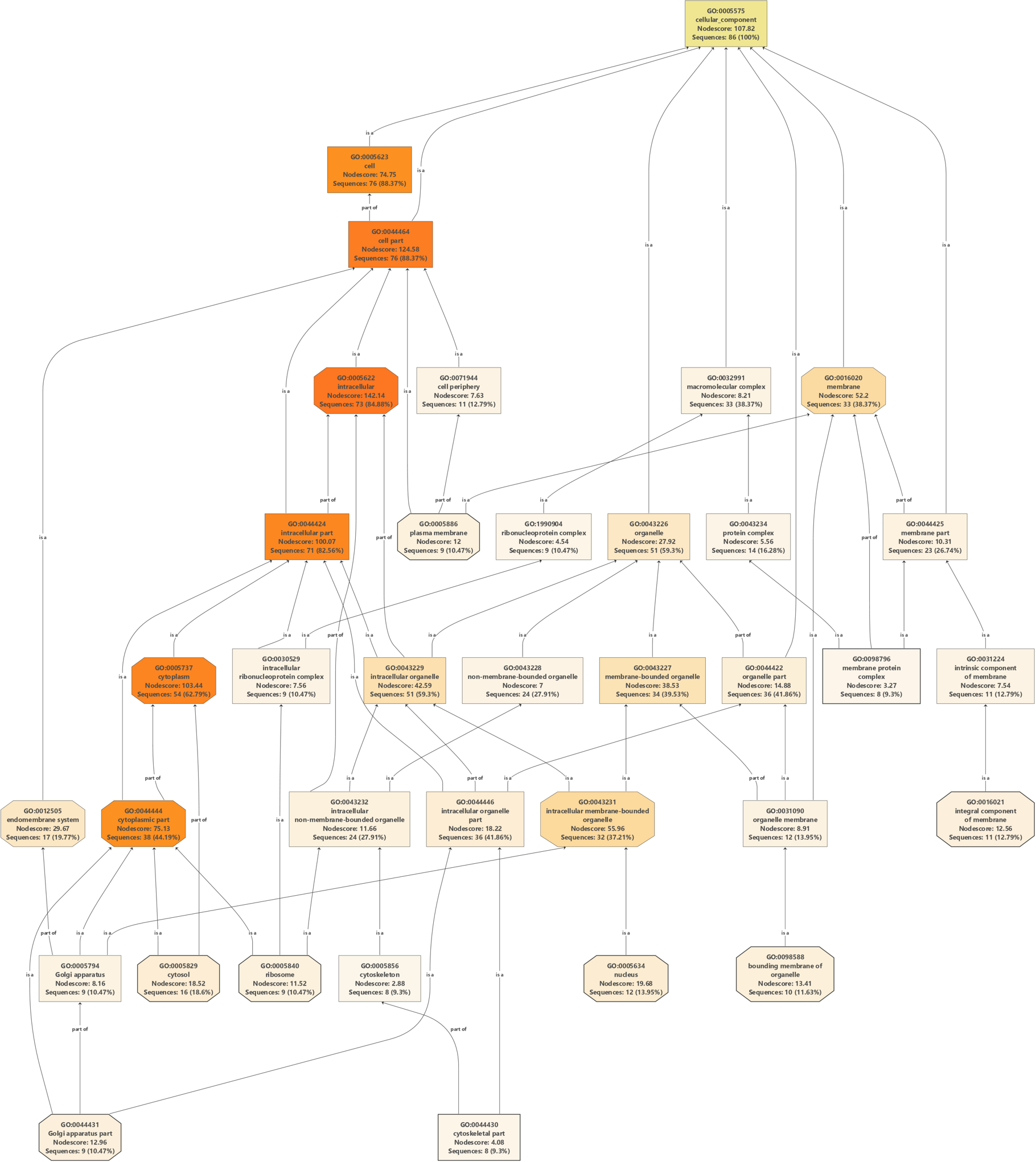

Supplement: Supplementary file 1 [file marinedrugs-16-00042-s001.zip › Figure S2.pdf]
